# Circadian cardiac NAD^+^ metabolism, from transcriptional regulation to healthy aging

**DOI:** 10.1152/ajpcell.00239.2022

**Published:** 2022-09-05

**Authors:** Bryce J. Carpenter, Pieterjan Dierickx

**Affiliations:** ^1^Max Planck Institute for Heart and Lung Research, Bad Nauheim, Germany; ^2^Cardiopulmonary Institute (CPI), Bad Nauheim, Germany; ^3^German Centre for Cardiovascular Research (DZHK), Partner Site Rhine-Main, Bad Nauheim, Germany

**Keywords:** cardiovascular health, circadian rhythms, NAD^+^ metabolism

## Abstract

Nicotinamide adenine dinucleotide (NAD^+^) is a critical redox factor and coenzyme with rhythmic availability, and reduced NAD^+^ levels are a common factor in many disease states, including risk factors associated with aging. Recent studies have expanded on the role of circadian rhythms and the core clock factors that maintain them in the regulation of NAD^+^ levels in the heart. This has revealed that NAD^+^ pools and their use are tightly linked to cardiac function, but also heart failure. The convergence of these fields, namely, clock regulation, heart disease, and NAD^+^ metabolism present a complex network ripe with potential scientific and clinical discoveries, given the growing number of animal models, recently developed technology, and opportunity for safe and accessible precursor supplementation. This review seeks to briefly present known information on circadian rhythms in the heart, connect that research to our understanding of cardiac NAD^+^ metabolism, and finally discuss potential future experiments to better understand interventional opportunities in cardiovascular health regarding these subjects.

## Graphical abstract

**Figure:**
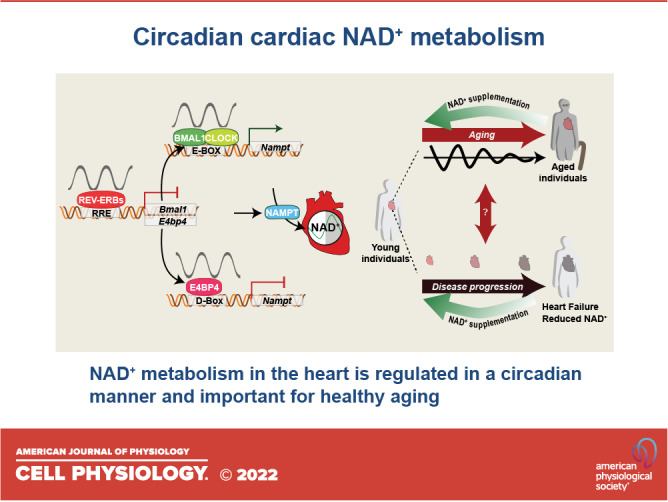

## INTRODUCTION

Circadian rhythms, the roughly 24-h internal cycles used by nearly all known life, are recognized to synchronize a wide array of biological processes. This synchronization is necessary for the effective utilization of limited cellular resources, such as mobilizing metabolites before the need for them in anticipation of environmental variations such as light/dark and feeding/fasting cycles (1). Diurnal rhythms are maintained by a feedback loop of core clock components that regulate the expression of other clock pathway components and clock-controlled output genes. These clock-controlled genes are regulated in a tissue and cell type-specific manner (2), driving rhythmic physiological processes such as de novo lipogenesis in the liver (3) or lipolysis and thermogenesis in adipose tissue (4). The heart presents an interesting case in that it relies on high energy expenditure to maintain functions critical to acute and constitutive health such as blood pressure, heart rate, and vascular status, all demonstrating daily rhythmicity (5–7). This is in part driven by a network of metabolic genes, proteins, and coenzymes that oscillate. Nicotinamide adenine dinucleotide (NAD^+^), a redox factor in the production of ATP and a coenzyme to hundreds of enzymes (8), is one such factor that is rhythmically regulated. There have been incredible bouts of progress on the study and supplementation of NAD^+^ in disease and aging states over the last several years, particularly in relation to its circadian distribution and role in preventing or mitigating cardiovascular injury. Sleep/wake cycles, body temperature, and blood glucose are all examples of outputs that shift in period or decrease in rhythmic amplitude when comparing older human and mouse cohorts to younger groups (9), and NAD^+^ levels may also change with age. There is evidence that even some core clock components exhibit dampened rhythmicity in older subjects (10), providing a potential mechanism for the changes listed earlier. Circadian dysregulation is associated with an increased risk of cardiovascular disease, seen in examples like shiftwork and obesity (11). Significant cardiac events are even much more likely to happen shortly after the onset of the active phase when cortisol is at its peak (5). Reduced NAD^+^ levels are also correlated with severe heart disease (12), though the precise pathology is still being elucidated. This review will seek to establish what is currently known about the interaction of core clock components with NAD^+^ metabolism in the mammalian heart, as well as highlight some promising recent studies in the usage of NAD^+^ supplements to treat aging and heart failure with the clock in mind.

## CLOCK FACTOR DISRUPTION RESULTS IN CARDIAC DYSFUNCTION DUE TO DEREGULATED METABOLISM

The clock is driven by a feedback loop of transcriptional activators and repressors that regulate the expression of each other, carefully synchronized by environmental cues like light or feeding [extensively reviewed by Guan et al. (13)]. The most redundantly and robustly regulated member of the mammalian core clock components is *Arntl* (*Bmal1*), which encodes the protein: brain and muscle ARNT-like 1 (BMAL1). BMAL1 heterodimerizes with circadian locomotor output cycles kaput (CLOCK) to form a transcriptional activating complex, whereas nuclear receptors REV-ERBα and REV-ERBβ repress the expression of *Bmal1* (14). Via epigenomic regulation through recruitment of Nuclear Receptor CoRepressor 1 (NCoR1) and Histone Deacetylase 3 (HDAC3) (15, 16), REV-ERBs also contribute to the transcriptional repression of many clock-controlled genes such as important metabolic factors (17). Although REV-ERBs have been well-defined in several tissues, particularly liver and adipose (17), the role of REV-ERBs in the heart has only just begun to be studied (18–20). REV-ERBs, like other clock factors, would be expected to carry critical importance in the heart, given the reliance of the heart on fatty acid oxidation and the well-documented control of lipid metabolism by REV-ERBs in other tissues. Despite the united primary classification of “molecular core clock components,” these proteins display significant structural and functional differences. Together, the core clock factors maintain an intricate network of activating and repressing transcription factors and enzymes involved in posttranslational modification of histones and other proteins.

The interdependency of this system presents a unique challenge to the analysis and interpretation of results, as modifying one clock component will change the expression of others. In addition, circadian rhythmicity is not a variable that can be fully isolated from the multifunctional factors that constitute the core clock, each with their own contribution to the core feedback loop and other molecular roles. A variety of models have been generated, predominantly in mice, to better define these pairings of particular proteins to distinct roles, as well as exploring the mechanism by which the same factor can have different roles between different tissues (2). Although there are a number of models to simulate physiological clock disruption via the use of environmental disruption (e.g., light/feeding regiments), genetic deletion (knockout, KO) models have been the most successful in demonstrating the cardiac-specific purposes for each protein of interest.

A whole body KO of *Bmal1* in mice completely ablates rhythmicity of the central and peripheral clocks, and these mice present a host of disease phenotypes: muscular atrophy, substantial reduction in weight gain, impaired metabolism, dilated cardiomyopathy, and advanced aging, culminating in a greatly reduced lifespan (21). As BMAL1 ablation has been previously shown to act as the lynchpin of the clock, this highlights the critical importance of total circadian rhythms to organismal physiology. However, a conditional knockout of *Bmal1* in cardiomyocytes recapitulates the age-progressed dilated cardiomyopathy (DCM) of the whole body knockout, and further isolates the role of BMAL1 in regulating cardiac energy supply directly through activating expression of other factors of signaling and metabolism (22, 23). Much like the cardiomyocyte-specific *Bmal1* knockout, cardiomyocyte-specific *Reverbα/β* deletions lead to dilated cardiomyopathy and impaired metabolism in the mouse heart, eventually resulting in premature death (18, 19). The feedback loop between BMAL1 and REV-ERBα/β means that the knockout of *Bmal1* has considerable downregulation of *Rev-erbs* (22), whereas the genetic double knockout of *Rev-erbα/β* leads to constant, high expression of *Bmal1* (18). These confounding expression effects are one potential explanation, along with the additional complication of rhythmic disruption, to the fact that these genetic models may share phenotypes. In addition, these factors may coordinate or compete at multiple levels of control. In both the *Bmal1* and *Rev-erbα/β* knockouts, with very low and very high levels of BMAL1, respectively, the expression of *Nampt*, the rate-limiting enzyme in NAD^+^ biosynthesis (24), is heavily reduced (18, 22). Thus, seemingly opposed models of circadian factor deletion can instigate the same metabolic defects.

CLOCK functions in the heterodimer with BMAL1 as a transcriptional activator through binding to E-box motifs at cis-regulatory elements and as a histone acetyltransferase (HAT) to open chromatin, allowing for transcription (25, 26). A cardiomyocyte-specific CLOCK dominant-negative mutant holds some degree of similarity to the cardiomyocyte-specific *Bmal1* knockout, including hypertrophy, impaired metabolism, and loss of gene expression rhythmicity, but does not progress to the lethal dilated cardiomyopathy (22). These results highlight the necessity of different genetic clock mutant models to elucidate their similarities, but more so their intricate differences. Regardless, the acetylation activity of CLOCK is another mechanism by which clock factors can impact the attribution of cellular resources.

In addition, many of these proteins are believed to acquire new roles in the context of tissue development and aging, introducing a further level of complication between developmental and inducible knockout models. An inducible model of *Bmal1* whole body knockout in 12-wk-old mice still disables behavioral and transcriptional output of the clock, without reducing lifespan or affecting metabolism to a significant degree (27). An inducible, cardiomyocyte-specific deletion of *Bmal1* in adult mice demonstrated a susceptibility to arrythmia in addition to a globally reduced heart rate (28), proposed to be managed through ion channel control (29). However, there is currently no published study to investigate the long-term survival of these mice, as the study mentioned earlier only included mice up to 20-wk old, whereas the study of the conditional cardiomyocyte-specific *Bmal1* deletion only began to reveal major heart defects, such as reduced ejection fraction and fractional shortening, at the 20-wk mark (22). To the best of our knowledge, published studies are currently limited to inducible KO of only *Bmal1* so far, though they still mark a potential important distinction in the critical developmental role of BMAL1. Similar and more thorough studies of other circadian clock factors, such as the REV-ERBs, are likely necessary to understand the importance of rhythms during organism development. In line, human embryonic stem cells in vitro lack rhythmic expression of clock factors before gaining rhythmicity during differentiation into cardiomyocytes suggesting a role of the clock during cell type specification (6). The combination of less severe phenotypes in inducible mouse studies and the lack of rhythmic expression in human embryonic cells implies there could even be noncircadian functions of clock factors, further distinguishing the individual roles each factor could have. However, further experiments and additional models are needed to test this hypothesis. The considerable difficulty of longitudinal short-term sampling needed to assess circadian data, a challenge exacerbated in the heart particularly, has largely precluded interventional study in humans. Cardiomyocytes derived from human embryonic stem cells have preliminarily supported the importance of the clock in human hearts, especially in stress response (6). Relatedly, REV-ERBα was identified in human heart biopsy transcriptomics as a potential mediator of the diurnal variation in perioperative myocardial injury (30). On the other end of the lifespan, analysis of circadian factor mRNA expression in 22-mo-old versus 3-mo-old mice found tissue-specific alterations in acrophase and rhythm amplitude. *Bmal1* and *Rev-erbα* both retained rhythmicity and phase in the older hearts but did decrease in amplitude (10). This effect remains to be confirmed at the protein level, but further confirms a relationship between dampening rhythms and aging, especially as the heart loses healthy variation in function with age (31). Whether regular aging leads to circadian dampening or dysfunction of circadian factors leads to the other hallmarks of aging, as seen in the knockout models, is unclear. However, much work remains in developing models that accurately accommodate the fine-tuned conditions of circadian factors, such as age and tissue environment (32).

## NAD^+^ METABOLISM AND CIRCADIAN REGULATION

**Figure 1.**
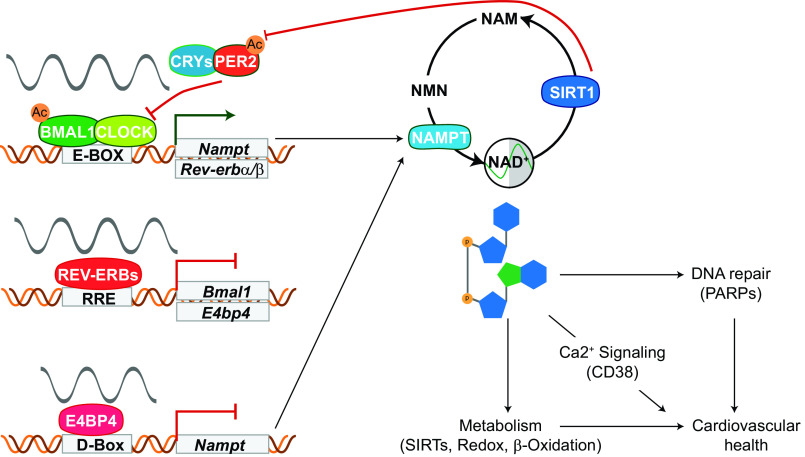
Expression of *Nampt* is under rhythmic control of clock factors, whereas NAD^+^ is consumed to inhibit clock factor activity and support downstream metabolic processes that promote cardiovascular health. BMAL1:CLOCK and REV-ERBs via E4BP4 coordinate transcriptional control of *Nampt*, the rate-limiting enzyme in NAD^+^ synthesis and salvage. SIRT1 is an NAD^+^-dependent deacetylase that acts on both BMAL1 and PER2, repressing transcription of *Nampt*. SIRT1 produces and is inhibited by NAM, providing an additional layer of NAD^+^ cyclic control through precursor availability and regulation of consumption. BMAL1, brain and muscle ARNT-like 1; CLOCK, circadian locomotor output cycles kaput; NAD^+^, nicotinamide adenine dinucleotide; NAM, nicotinamide; NAMPT, nicotinamide phosphoribosyltransferase; NMN, nicotinamide mononucleotide; PARP, poly-[ADP-ribose] polymerase; SIRTs, Sirtuins.

NAD^+^ level alterations are a persistent finding in manipulation of clock components in the heart. NAD^+^ levels have been confirmed to rhythmically vary in wild-type conditions over the course of a day (18, 33), seemingly related to the rhythmic control of nicotinamide phosphoribosyltransferase (NAMPT). Several tissues, including the heart, rely primarily on the conversion of nicotinamide (NAM) leftover from NAD^+^ usage into nicotinamide mononucleotide (NMN) by NAMPT to preserve functional stores. Prior evidence has shown that the BMAL1:CLOCK heterodimer activates *Nampt* expression directly (25; Fig. 1). REV-ERBs protect *Nampt* expression by repressing *E4bp4*, another transcriptional repressor that was only very recently found to target *Nampt* in the heart (18; Fig. 1), but has previously been implicated in heart health (34, 35). The effect of E4BP4 repression on *Nampt* appears to be dominant to activation by the BMAL1:CLOCK heterodimer, given that the reduction persists through the induction of BMAL1 in the *Rev-erbα/β* knockout (18). It is likely the low levels of NAD^+^ contribute to the lethality of the phenotype in the constitutive knockout models of either *Bmal1* or the *Rev-erb*s (36). Though *Nampt* is clearly regulated by clock factors, NAMPT protein expression is much more stable (37), and NAD^+^ levels continue to oscillate even during constitutively low NAMPT levels (18). These results reveal that additional factors beyond NAMPT-mediated regulation contribute to rhythmic NAD^+^ levels and suggest that multiple mechanisms are at play depending on the context and time of the day.

Indeed, NAMPT is not the only method to generate NAD^+^. NMN can also be produced from nicotinamide riboside (NR), available from diet, through NR kinases (NMRKs; 38). The combination of processes managed by NMRKs and NAMPT to produce NMN, followed by the transformation of NMN into NAD^+^ by nicotinamide-nucleotide adenylyltransferases (NMNATs), account for the amidated production pathways that provide over 99% of available NAD^+^ in the heart (38). The rhythmic presence and activity of these proteins are not sufficiently studied to understand how they may contribute to the persistent rhythmicity of the NAD^+^ pool when NAMPT is reduced, though NMRK2 has been shown to remain or increase in contexts where NAMPT has waned, like aging or heart failure (39). The rhythmicity of total NAD^+^ may still just be driven by other behavioral rhythms such as feeding or locomotor activity, though there currently exist no studies the authors could find that definitively expose the contributions of such circadian outputs to the daily rhythmicity of cardiovascular NAD^+^ pools. However, mouse models involving some forms of diet intervention like high-fat diet can impact the rhythmicity of circadian-controlled factors (40), whereas caloric restriction interventions have shown a capacity to modulate NAD^+^ and mitochondrial factors in the liver as a response to nutrition dynamics (41). Though these studies focused on the liver, the sensitivity of the heart to oxidative stress and fuel availability indicates such drastic metabolic changes are likely to impact the heart (42). Importantly, caloric restriction has been suggested to ameliorate cardiovascular aging through reducing oxidative stress (43), and increasing available free fatty acids with high-fat, high-sucrose diet was recently reported to temporarily protect mice from heart failure induced by deletion of REV-ERBs (19). NAD^+^ is also understood to fall under spatial regulation between mitochondrial and cytosolic portions, so rhythms may vary or depend on the transport of precursors and synthesis enzymes between organelles (12). Beyond synthesis or diet, the use of NAD^+^ as a consumable metabolite in enzymatic reactions and as a redox factor could also direct rhythmic status of the NAD^+^ pool in quantity and cellular location. There are several examples of enzymes that use NAD^+^ for their activity, often resulting in NAM to be salvaged, that may also interact with core clock components. Although many of these precise relationships remain to be demonstrated conclusively in the heart, there are already significant implications for the importance of these enzymes in cardiovascular health and their reliance on NAD^+^. Sirtuins (SIRTs) are class III deacetylases that use energy from the breakdown of NAD^+^ into NAM to deacetylate proteins, including histones, and are simultaneously inhibited by NAM (44, 45). Thus, in a manner reminiscent of circadian rhythms, they participate in their own regulation. SIRT1 is a nuclear sirtuin that impacts the clock by directly deacetylating BMAL1 (25), impairing its ability to activate, and deacetylating PER2, an important repressor of BMAL1 (Fig. 1). Increased acetylation of PER2 increases the time PER2 spends in the nucleus, impeding BMAL1 activity (46).

Properly regulated *Sirt1* expression and activity has been repeatedly demonstrated to play a major role in heart development (47), healthy cardiac function (48), and response to various forms of cardiac injury (44, 48). A heart-specific *Sirt1* deletion imparts mitochondrial dysfunction and progresses to dilated cardiomyopathy in adulthood (47), whereas a deletion induced at 8 wk of age develops a relatively minor decrease in heart function only 11 mo after deletion (48). However, *Sirt1* expression has previously been shown to decrease rapidly shortly after birth and to decrease further in aged mice (49), underscoring the role of SIRT1 in development and stress response. Indeed, the inducible deletion model was much more susceptible to pressure overload caused by transverse aortic constriction (48), whereas activation and overexpression of *Sirt1* mitigates damage from ischemia/reperfusion injury (50). Leukocytes (51) and biopsies of the left atrial myocardium derived from human patients have suggested that SIRT1 protein is downregulated in heart failure (49), corresponding with increased oxidative stress and apoptosis. These processes are at least partially mediated by the deacetylation of Forkhead box O (FOXO) transcription factors and p53 (50). Because NAD^+^ is critical for SIRT1 activity, this provides a downstream manner for NAD^+^ to affect heart health in both progressive and acute situations.

One of the most well-understood functions of NAD^+^ is its biochemical role in the transfer of electrons during ATP production. On accepting an electron, NAD^+^ is reduced to NADH, a distinct molecule that cannot be used in place of NAD^+^, despite their similarities. The conversion of NAD^+^ into NADH occurs three times in one turn of the Krebs cycle, as well as once for each conversion of glyceraldehyde-3-P into pyruvate and once during conversion of pyruvate into acetyl-CoA (52). NAD^+^ is also reduced once during β-oxidation and once during ketogenesis. NADH then oxidizes back into NAD^+^ by donating the electron during cytosolic lactic acid fermentation or at mitochondrial Complex 1 of the electron transport chain (52). Because of the necessity of both molecules and their shared components, both NAD^+^ and NADH must be present at sufficient quantities and maintain a ratio reflecting redox status. An imbalance of these metabolic processes can result in host of issues, including an overproduction of reactive oxygen species in the mitochondria (53). This fluctuation of NAD^+^/NADH ratio can be perturbed by disruption of the enzymes mediating the process, such as lactate dehydrogenase in the cytosol or the mitochondrial complex members (53, 54), or from a nutrition imbalance like hyperlipidemia, common to diabetes (42). Conversely, dysfunction of NAD^+^/NADH allocation between cellular regions or redox state may impair energy production for the cell, clearly impacting the health of the tissue. Hyperacetylation of mitochondrial transport members or products due to impaired sirtuin activity may also exacerbate the imbalance, particularly since sirtuins rely on available NAD^+^ and are inhibited by NADH (54, 55). Although hyperacetylation from sirtuin inhibition and increased amounts of reactive oxygen species can damage the mitochondria and ability of the heart to produce energy, NADH prevented from donating the electron due to locked metabolism also means that the NAD^+^ pool available for other enzymes will be reduced, further complicating the situation in heart disease. Glycolysis and nutrient availability to the heart are governed by circadian rhythms, providing another avenue for the perturbation of the crucial balance of NAD^+^ and NADH.

As seen in clock factors and circadian rhythms themselves, NAD^+^ levels vary with regards to organism age (8, 56). Age is strongly associated with changes in clock outputs, such as feeding parameters and sleep/wake cycles, for both humans and mice (5, 9, 31, 57). Sufficient NAD^+^ is also heavily implicated in healthy aging, as many tissues show lower NAD^+^ levels in aged cohorts (8, 58), and boosted NAD^+^ levels correlate with longer life and better tissue function (59). However, there is not sufficient evidence that the heart decreases in NAD^+^ stores simply by consequence of age (58, 60), though the risk of heart failure does increase with age (12). There are also NAD^+^-dependent enzymes with functions in the heart that may have increased activation with aging, such as poly-[ADP-ribose] polymerase 1 (PARP1) in DNA repair (61) or cyclic ADP ribose hydrolase (CD38) in calcium signaling and inflammation (62, 63; Fig. 1). Reduced NAD^+^ levels are a common hallmark of critical heart diseases such as dilated cardiomyopathy (DCM) and cardiac ischemia/reperfusion (I/R; 12, 39, 50). Several models of clock factor mutations in mice demonstrate advanced aging phenotypes, including a propensity for heart disease and impaired NAD^+^ metabolism (8, 18, 22). Given the evidence displayed earlier, deregulation of *Nampt* from disruption of circadian factors has been identified as a potential cause for reduced levels of NAD^+^, and *Nampt* expression has been found to decrease with age and in heart failure models (12, 39). Strikingly, *Nmrk2* was found to be largely upregulated in mouse models of heart failure and persistent through wild-type aging, unlike *Nampt*, *Nmrk1*, and *Sirt1* (39). At 24-mo old, cardiac *Nmrk2* deficient mice were no longer able to maintain regular NAD^+^ levels (39). Although it is very difficult to parse the cause-and-effect relationships between circadian disruption/dampening, aging, and impaired NAD^+^ metabolism, the current state of research has made the restoration of NAD^+^ a clear target for potentially ameliorating pathological symptoms across these disease states.

## NAD^+^ SUPPLEMENTATION OF THE HEART IN CARDIOVASCULAR DISEASE

**Figure 2.**
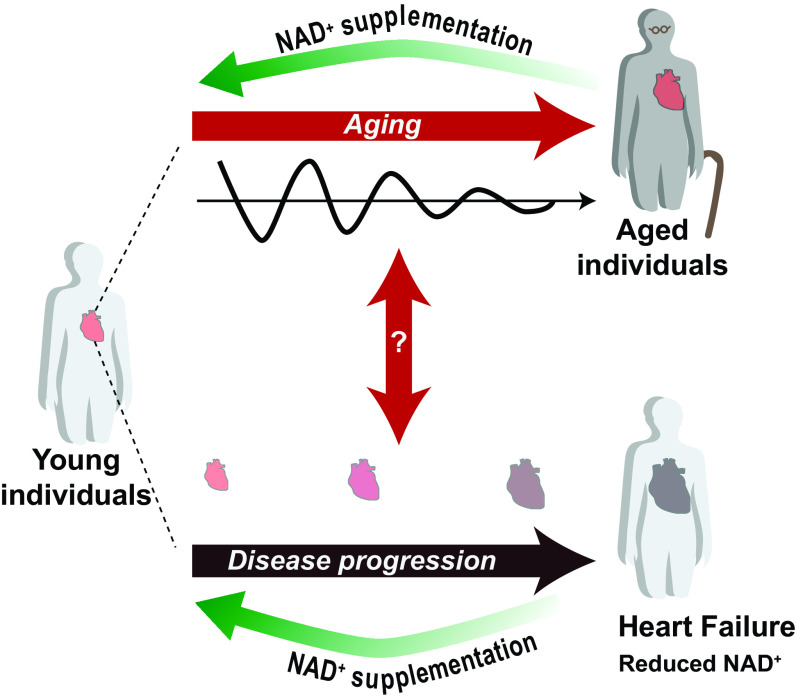
NAD^+^ metabolism as a central component in the relationship between aging, dampening circadian rhythms, and heart disease progression. NAD^+^ supplementation has been suggested as an antiaging strategy for some time and has recently seen expanded use in treating heart disease and injury. However, the circadian aspect of NAD^+^ regulation suggests a new parameter to consider in delivery. New models and techniques accommodating this consideration will be necessary to interrogate the linkage between these disease factors and maximize treatment or prevention efficacy. NAD^+^, nicotinamide adenine dinucleotide.

As previously described, NAD^+^ is synthesized by a combination of different pathways, some redundant, and is utilized across many different tissues. In addition, just as every model of clock disruption depicts unique characteristics depending on which specific factor has been selected, different forms and causes of heart failure may have severely different and compounding etiologies (64). One major distinction is between heart failure with preserved ejection fraction (HFpEF) and heart failure with reduced ejection fraction (HFrEF), referring to the ability of the heart to effectively pump blood by contraction. The cross section of differing NAD^+^ metabolism deficiencies and various types of heart failure is thus very large, yet made all the more promising for therapeutic use for that very reason. In fact, niacin (NA) and NAM have been used historically in humans for the treatment of vitamin B3 deficiency (pellagra; 65), though their potential for treating heart failure has only been recognized for roughly two decades now (Fig. 2). It is important to remember that the vast majority of cardiac NAD^+^ is made through the amidated pathways, relying on salvage of NAM and dietary NR to make NMN before NAD^+^. Because NAD^+^ function is also dependent on subcellular localization after synthesis, there is a complex network of transporters to spatially regulate NAD^+^ and its precursors, including the only recently discovered mammalian SLC25A51 for the import of NAD^+^ directly into mitochondria (66, 67).

Encouragingly, there are many mouse models that have successfully managed to increase NAD^+^ pools to combat heart failure (12, 36, 65, 68, 69). Overexpression of *Nampt* and supplementation with precursors NAM, NR, and NMN have already been demonstrated to improve cardiac function and defend against cardiac injury in several forms of heart disease (12, 65, 68). Unfortunately, the variability in disease states, examined parameters, and delivery strategies have prevented the conclusion of distinct, optimal treatment options. Often, the critical readout of actual NAD^+^ levels has been left out of these studies, or the increase does not reach statistical significance, despite some clear effect on cardiac health (36, 68, 69). However, NAD^+^ does not exert its myriad effects solely through presence. The ratio of NAD^+^/NADH and the consumption of NAD^+^ in metabolism, protein modification, DNA repair, and other regulatory processes rely on the conversion of NAD^+^ into other forms. Thus, a measurement of flux between precursors, NAD^+^, and NADH may be more informative on supplementation efficacy, and NAD^+^ deficiency in heart disease than simple steady-state observations of NAD^+^ alone. Though potentially difficult or expensive to perform injudiciously, labeling studies for NAD^+^ synthesis have been successfully executed previously (57), and genetic tools have been made to successfully distinguish cytosolic and mitochondrial measurements of NAD^+^ and NADH in isolated cardiomyocytes (70). In the case of NR, there is a significant clinical appeal in its oral availability (71), but investigations into muscle and heart NR supplementation find that much of the orally given NR is absorbed by the liver (36, 72, 73). Intraperitoneal supplementation with NR in a nondisease state reduced *Nmrk2* expression (72), which may explain why some heart studies were unable to elevate NAD^+^ levels with NR in control mice (69). NMN is most often tried in mouse models with intraperitoneal injections, but toxicity quantities have not been evaluated sufficiently in humans (65, 68).

Dilated cardiomyopathy with reduced ejection fraction is a broad form of heart failure in humans, with many varying causes such as diabetes, alcohol excess, and ischemic cardiomyopathies, among others. As this symptomology is frequently characterized by reduced *Nampt* expression in relevant mouse models (65), NAM is a suboptimal choice for supplementation here. NAD^+^ may still be generated by remaining NAMPT in these models, but the turnover rate will be largely decreased, whereas the larger NAM reserve continues to inhibit sirtuins (44). Despite the fresh discovery of SLC25A51 as a transporter of NAD^+^ into mitochondria for mammals (66, 67), prior evidence still supports that NAD^+^ must be cleaved into smaller precursors to enter the cell in the first place (12). Thus, NR and NMN remain the most efficient methods to effectively boost cardiac NAD^+^ levels. In the instance of DCM marked by reduced *Nampt* and induced *Nmrk2* (69), NR may be the more promising option for humans, corroborated by the extensive research that has been done on oral supplementation of NR (36, 65, 71). NR supplementation via the drinking water has prolonged the survival of female mice suffering from dilated cardiomyopathy and HFrEF due to disruption of REV-ERBs (36) and has yet to be tested in other circadian clock mutants. Although not completely able to rescue this drastic phenotype, these data do underscore the impact of NAD^+^ reduction on lethality and offers hope of affecting a wide variety of other models. In addition, this experiment supplied NR constitutively, potentially perturbing the natural physiological circadian regulation of NAD^+^ on the other end of the spectrum, whereas a model with rhythmic supplementation may impact the delivery and efficacy of the treatment to a greater extent (32). This question pertains to humans as well, as initial clinical trials to improve mitochondrial function in skeletal muscle with NR showed minimal changes in target parameters, though the treatment also failed to induce any increase to NAD^+^ in the skeletal muscle (74, 75). However, both preclinical and clinical trials have had varied success over different tissues and disease states (76), and there have already been two human clinical trials centered around heart disease that have successfully raised blood NA^+^D^+^ levels and ameliorated some parameters of heart disease (77, 78). Recently completed human trials on the safety of NR supplementation in patients with HFrEF found oral delivery of NR to be safe and effective at raising blood NAD^+^ levels (79), with future investigations sure to follow shortly.

## CONCLUSIONS

Undoubtedly, the impact of circadian rhythms on metabolism underscores an important role the molecular clock plays in maintaining cardiovascular health, and may well be key to lessening the costs of cardiovascular disease (Fig. 1). The circadian regulation of *Nampt* by the repressor E4BP4 downstream of repressive REV-ERBs (18), mirrored by the transcriptional activating complex of BMAL1:CLOCK also regulating *Nampt* expression (22), links the common feature of decreased NAD^+^ in heart failure to circadian disruption and aging. However, many questions still require resolution in these respects, necessitating newer models and further questions. In particular, the novel finding that E4BP4 represses *Nampt* in the heart accentuates the need for a proper characterization of a cardiac-specific KO of *E4bp4*, given that prior evidence has shown overexpression of E4BP4 protein in diseased heart tissue (35). The flow and causational relationship between these factors, such as whether dampened rhythmicity induces aging (10) or if it is the other way around, or whether NAD^+^ reduction is a symptom or pathology to heart failure, also confounds the potential for treatment (Fig. 2). Despite very recent and significant progress in our understanding of NAD^+^ synthesis and the role such an important metabolite holds in the heart, more studies are needed to standardize work in the field and begin applying this knowledge to humans. NAD^+^ precursors and their dynamics must be investigated further to use them safely and effectively in treating heart failure and aging, and this work should be prioritized given the already very promising rescue experiments performed in mouse models. Thankfully, recent upgrades to technology such as labeling capabilities, in vivo imaging, and sensitive biosensors should prove to be a massive boon to the study of NAD^+^ precursor involvement and distribution. Our understanding of NAD^+^ as a circadian metabolite is also continuing to advance, and ignoring the natural rhythmicity of such a critical factor could easily impair the medical efficacy of the precursors as treatment options. As the field expands on the knowledge of effective delivery methods, further detail should be given to the possible effects of temporal distribution of the drug of choice. Finally, though NAD^+^ has frequently been studied in the context of aging, preparing a proper method of delivery and choice of precursor could make massive strides in connecting the role of aging in the heart to NAD^+^ specifically, and the shifts and modulations of circadian rhythms in NAD^+^ metabolism, aging, and cardiovascular disease should not be ignored.

## GRANTS

This work was supported by the DFG [Excellence Cluster Cardio-Pulmonary Institute (CPI)].

## DISCLOSURES

No conflicts of interest, financial or otherwise, are declared by the authors.

## AUTHOR CONTRIBUTIONS

B.J.C. and P.D. conceived and designed research; prepared figures; drafted manuscript; edited and revised manuscript; approved final version of manuscript.
